# Lactotransferrin Downregulation Serves as a Potential Predictor for the Therapeutic Effectiveness of mTOR Inhibitors in the Metastatic Clear Cell Renal Cell Carcinoma without *PTEN* Mutation

**DOI:** 10.3390/biomedicines9121896

**Published:** 2021-12-13

**Authors:** Jing-Quan Zheng, Che-Hsuan Lin, Hsun-Hua Lee, Wen-Ke Wang, Yiu-Shun Tong, Kang-Yun Lee, Hui-Wen Chiu, Yuan-Feng Lin

**Affiliations:** 1 Graduate Institute of Clinical Medicine, College of Medicine, Taipei Medical University, Taipei 11031, Taiwan; d118106006@tmu.edu.tw (J.-Q.Z.); d118108003@tmu.edu.tw (W.-K.W.); biemtong@tmu.edu.tw (Y.-S.T.); 13258@s.tmu.edu.tw (K.-Y.L.); 2 Division of Pulmonary Medicine, Department of Internal Medicine, Shuang Ho Hospital, Taipei Medical University, New Taipei City 23561, Taiwan; 3 Division of Pulmonary Medicine, Department of Internal Medicine, School of Medicine, College of Medicine, Taipei Medical University, Taipei 11031, Taiwan; 4 Department of Otolaryngology, Taipei Medical University Hospital, Taipei Medical University, Taipei 11031, Taiwan; d119105002@tmu.edu.tw; 5 Department of Otolaryngology, School of Medicine, College of Medicine, Taipei Medical University, Taipei 11031, Taiwan; 6 Department of Neurology, Shuang Ho Hospital, Taipei Medical University, New Taipei City 23561, Taiwan; kaorulei@tmu.edu.tw; 7 Department of Neurology, School of Medicine, College of Medicine, Taipei Medical University, Taipei 11031, Taiwan; 8 Vertigo and Balance Impairment Center, Department of Neurology, Shuang Ho Hospital, Taipei Medical University, New Taipei City 23561, Taiwan; 9 Department of Surgery, Taipei Medical University Hospital, Taipei Medical University, Taipei 11031, Taiwan; 10 Department of Medical Research, Shuang Ho Hospital, Taipei Medical University, New Taipei City 23561, Taiwan; 11 TMU Research Center of Urology and Kidney, Taipei Medical University, Taipei 11031, Taiwan; 12 Cell Physiology and Molecular Image Research Center, Wan Fang Hospital, Taipei Medical University, Taipei 11696, Taiwan

**Keywords:** renal cell carcinoma, LTF, mTOR, autophagy, metastasis

## Abstract

Approximately 30% of clear cell renal cell carcinoma (ccRCC) patients develop metastatic spread at the first diagnosis. Therefore, identifying a useful biomarker to predict ccRCC metastasis or therapeutic effectiveness in ccRCC patients is urgently needed. Previously, we demonstrated that lactotransferrin (LTF) downregulation enhanced the metastatic potential of ccRCC. Here, we show that LTF expression conversely associates with the mTORC1 activity as simulated by gene set enrichment analysis (GSEA). Moreover, Western blot analyses revealed that the LTF knockdown promoted, but the inclusion of recombinant human LTF protein suppressed, the phosphorylation of Akt/mTOR proteins in the detected ccRCC cells. Kaplan–Meier analyses demonstrated that the signature of combining an upregulated mTORC1 activity with a downregulated LTF expression referred to a worse overall and progression-free survival probabilities and associated with distant cancer metastasis in TCGA ccRCC patients. Furthermore, we found that the LTF-suppressed Akt/mTOR activation triggered an increased formation of autophagy in the highly metastatic ccRCC cells. The addition of autophagy inhibitor 3-methyadenine restored the LTF-suppressed cellular migration ability of highly metastatic ccRCC cells. Receiver operating characteristic (ROC) analyses showed that the expression of the LTF and MTORC1 gene set, not the autophagy gene set, could be the useful biomarkers to predict 5-year overall survival rate and cancer progression in ccRCC patients. Significantly, the signature of combining mTORC1 upregulation and LTF downregulation was shown as an independent prognostic factor in a multivariate analysis under the progression-free survival condition using the TCGA ccRCC database. Finally, the treatment with mTOR inhibitor rapamycin predominantly reduced the formation of autophagy and ultimately mitigated the cellular migration ability of ccRCC cells with LTF knockdown. Our findings suggest that LTF downregulation is a biomarker for guiding the use of mTOR inhibitors to combat metastatic ccRCC in the clinic.

## 1. Introduction

Renal cell carcinoma (RCC) accounts for approximately 90% of kidney cancers and is classified into three major subtypes clear cell RCC (ccRCC), papillary RCC (pRCC) and chromophobe RCC (chRCC). ccRCC is the main subtype (>75%) and correlates with the leading cause of deaths in patients with kidney cancer [1]. Approximately 30% of ccRCC patients with localized disease ultimately develop distant metastases after nephrectomy, which are linked to a high risk of mortality [2]. Recently, receptor tyrosine kinase inhibitors (TKIs) pazopanib and sunitinib have been used to treat metastatic ccRCC via suppressing the function of vascular endothelial growth factor and mechanistic target of rapamycin (mTOR), respectively. However, the therapeutic effectiveness of these TKIs on metastatic ccRCC still needs to be improved [3]. As a result, identifying a useful biomarker is urgently needed to guide the administration of TKIs in combating metastatic ccRCC clinically.

Lactotransferrin (LTF), also name lactoferrin, was firstly detected in mammary secretions and then also reported to be synthesized by most mammalian tissues [4,5,6]. In addition to iron-binding protein, LTF has been thought to be a multifunctional protein [7]. Previous studies demonstrated that LTF possesses antifungal [8], antibacterial [9,10], antiviral [11,12], anti-inflammatory [13], immune regulatory activities [14,15] and anticancer properties [16,17,18]. The interaction of LTF and its receptors, such as LDL receptor-related protein 1 (LRP1/CD91) [19] and C-X-C-motif cytokine receptor 4 (CXCR4), has been shown to regulate several physiological activities. Recently, several lines of evidence have indicated that LTF has anticancer properties and suppresses cancer metastatic potentials. In breast cancer, LTF inhibited G1 cyclin-dependent kinases to trigger cell growth arrest [20] and selectively induced cell apoptosis of metastatic breast cancer cells [21]. The addition of LTF has also been found to reverse programming of epithelial-to-mesenchymal transition to mesenchymal-to-epithelial transition in oral squamous cell carcinoma [22]. Moreover, LTF appeared to suppress the cell growth of highly metastatic HT-29 colon cancer cells [23]. In our previous report, we have demonstrated that LTF downregulation forces the metastatic progression of ccRCC probably via activating several intracellular signaling cascades, e.g., the Akt/mTORC1 axis [24]. However, the comprehensive mechanism still needs to be further explored.

As a result, this study attempts to investigate the effect of LTF expression on the activation of Akt/mTOR signaling axis. We found that LTF knockdown promoted cellular migration ability and enhanced the activity of Akt/mTOR in the poorly metastatic ccRCC cells. Conversely, the inclusion of recombinant human LTF protein suppressed cell migration and the activation of Akt/mTOR in the highly metastatic ccRCC cells. Importantly, the combination of low-level LTF and high-level MTROC1 gene set expression strongly predicted a shortest time period for cancer progression after the first treatment in ccRCC patients. These findings suggest that LTF downregulation might be a useful biomarker to predict the therapeutic effectiveness of mTOR inhibitors on combating metastatic ccRCC in the clinic.

## 2. Materials and Methods

### 2.1. Clinical and Molecular Data for RCC Patients

All of clinical data for TCGA ccRCC patients, were collected from the UCSC Xena website (UCSC Xena. Available online: http://xena.ucsc.edu/welcome-to-ucsc-xena/, accessed on 1 February 2021). The transcriptional profiles obtained by RNAseq (polyA þ Illumina HiSeq, San Diego, CA, USA) experiments in TCGA ccRCC database were also downloaded from the UCSC Xena website.

### 2.2. Cell Culture

The human renal adenocarcinoma cell lines ACHN, Caki-1, 786-O and A498 were purchased from American Type Culture Collection (ATCC) and cultivated by the procedure according to the ATCC guideline. All cell culture components were purchased from Gibco Life Technologies (Thermo Fisher Scientific Inc., Waltham, MA, USA). The 293T cells were cultured in DMEM with 10% fetal bovine serum (FBS). Cells were incubated at 37 °C with 5% CO_2_.

### 2.3. Cellular Migration Assays

Boyden chamber trans-well assay (NeuroProbe, Gaithersburg, MD, USA) was used to detect cellular migration ability. Cells (1.5 × 10^4^) re-suspended in serum-free medium were seeded into the upper chamber of the device on a membrane (NeuroProbe) precoated with fibronectin (Sigma-Aldrich, Burlington, MA, USA), and the lower chamber was contained the conditioned medium. After a 3 h incubation, the membrane was soaked in methanol for 10 min and stained with Giemsa for 1 h. The membrane was then attached to slides. The cells on the upper side of the membrane were carefully removed with a cotton swab. The migrated cells were counted in three random areas under a microscope at 400× magnification.

### 2.4. Lentivirus Production and Transduction

Nonsilencing control and LTF shRNAs were purchased from the RNAi Core of Academia Sinica (Taipei, Taiwan) and then contransfected with envelope plasmids (pMD.G) and packaging plasmids (pCMV-dR8.91) of lentivirus into 293T cells. The lentiviral particles were harvested from the culture media posttransfection for 48 h. Green fluorescent protein (GFP) and GFP-LC3 fusion-expressing lentiviral particles were purchased from Cell Biolabs (San Diego, CA, USA). RCC cells were transduced with the lentiviruses at a multiplicity of infection of 10 in the presence of polybrene (Santa Cruz, Dallas, TX, USA) and selected using puromycin after infection to generate a stable clone.

### 2.5. Reverse Transcription PCR (RT-PCR)

Total RNA was extracted from the tested cells using a TRIzol extraction kit (Thermo Fisher Scientific Inc.). cDNA was converted from the aliquots (5 μg) of total RNA by M-MLV reverse transcriptase kit (Thermo Fisher Scientific Inc., Waltham, MA, USA) and then amplified with Taq-polymerase (Protech, Taipei, Taiwan) using paired primers (for LTF, forward-GACCTGTGGAAGGATATCTTGCTGTGGCGG and reverse-CACCGCCACAGCAAGATATCCTTCCACAGGTC; for GAPDH, forward-AGGTCGGAGTCAACGGATTTG and reverse-GTGATGGCATGGACTGTGGTC).

### 2.6. Dot Blot Assay

Aliquots of culture media (200 μL) were blotted on the nitrocellulose membrane by suction through the dot blot equipment. The membrane was then blocked with 5% bovine serum albumin (BSA) in the wash buffer (Tris-buffered saline (TBS) containing 0.1% Tween-20) overnight at 4 °C with a gentle agitation. After washing 3 times, the membrane was incubated with an LTF-specific antibody (Elabscience, Houston, Texas, USA) for 2 h at room temperature. The membrane was further incubated with a peroxidase-labeled secondary antibody for another 1 h at room temperature after several washing steps. The dot blots were visualized by using an enhanced chemiluminescence system (Amersham Biosciences, Tokyo, Japan).

### 2.7. Western Blotting Assay

Whole cell lysates (100 μg) from the designated experiments and TD-PM10315 TOOLS Pre-Stained Protein Marker (10–315 kDa) (BIOTOOLS Co., Ltd., Taipei, Taiwan) were loaded into each well of an SDS gel, separated by electrophoresis and then transferred to PVDF membranes. The membranes were incubated with blocking buffer (5% skim milk in TBS containing 0.1% Tween-20) for 2 h at room temperature. The samples were incubated with primary antibodies against phosphorylated Akt (p-Akt), Akt, p-mTOR, mTOR and LC3-I/II (Cell Signaling, Danvers, MA, USA), GAPDH (AbFrontier, Seoul, Korea) as well, overnight at 4 °C with gentle agitation. After several washes, the membranes were incubated with a peroxidase-labeled secondary antibody for 1 h at room temperature. Immunoreactive bands were analyzed by an enhanced chemiluminescence system (Amersham Biosciences, Tokyo, Japan). The original, uncropped and unadjusted images of Western blot analyses are shown in Supplementary Materials Figures S2 and S3.

### 2.8. Fluorescent Microscopic Observation

GFP- or GFP-LC3-expressing cells were cultivated in the serum-free media overnight prior to the addition of recombinant human LTF (Sino Biological Inc., Beijing, China) at 300 ng/mL. The punctate formation of autophagosomes in GFP-LC3-expressing cells was observed at the designated time points after the addition of LTF and counted from 5 different fields (>25 cells) in three independent experiments under a fluorescent microscope [25].

### 2.9. Statistical Analyses

Statistical analyses were performed by using SPSS 17.0 software (Informer Technologies, Roseau, Dominica). Spearman’s correlation test was performed to estimate the correlations between the expression of LTF and the autophagy gene set obtained from the Molecular Signatures Database (MSigDB, Available online: http://software.broadinstitute.org/gsea/msigdb, accessed on 1 February 2021) and to evaluate the coexpression of LTF with somatic genes in the TCGA ccRCC cohort. Survival probabilities were determined by a Kaplan–Meier analysis and log-rank tests. A nonparametric Mann–Whitney test and Kruskal–Wallis one-way ANOVA using a post hoc Dunn’s test was used to analyze data from 2 and more related samples, respectively. *p* values <0.05 in all analyses were considered statistically significant.

## 3. Results

### 3.1. LTF Downregulation Enhances mTORC1 Activity and Cellular Migration Ability and Associates with a Poor Prognosis in ccRCC Patients

**Figure 1:**
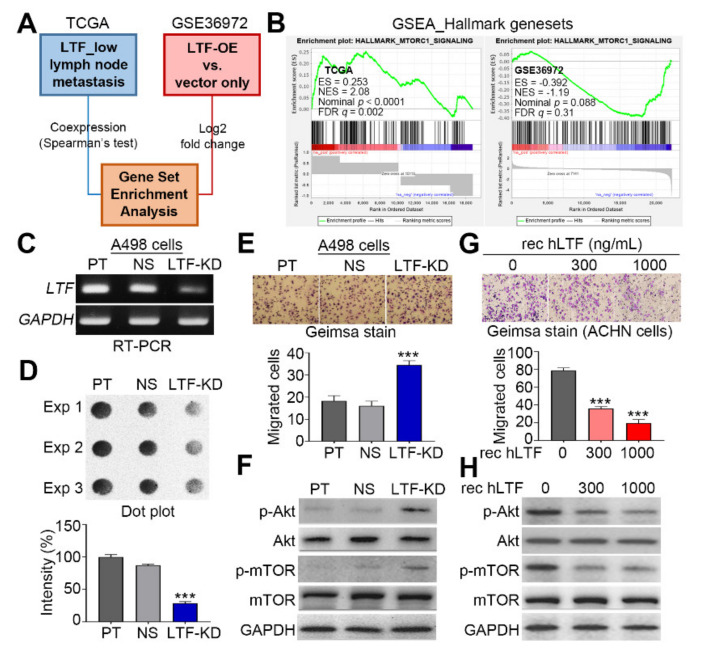
LTF expression negatively regulates the activity of mTORC1-related pathway and the cellular migration ability of ccRCC. (**A**) Flowchart for the generation of LTF-related gene signatures using TCGA ccRCC database and GSE36972 dataset in order to performing an in silico analysis using the GSEA program. (**B**) GSEA plots of Hallmark_mTOC1_signaling in the LTF coexpression signatures derived from metastatic ccRCC with low-level LTF (left) and LTF-overexpression cells (right). ES, NES and FDR denote enrichment score, normalized enrichment score and false discovery rate, respectively. (**C**,**D**) RT-PCR (**C**) and Dot blot (**D**) analyses for LTF mRNA and secreted protein levels, respectively, in parental (PT), nonsilencing (NS) control and LTF-knockdown (LTF-KD) A498 cells. GAPDH mRNA levels were used as an internal control for RT-PCR experiment. (**E**) Giemsa staining (top) and data from three independent experiments (bottom) for the migrated cells in the 3 h transwell assay for the A498 cell variants. (**F**) Western blot analysis for the protein levels of phosphorylated Akt (p-Akt), Akt, p-mTOR, mTOR and GAPDH in the indicated A498 cell variants. (**G**) Giemsa staining for the migrated cells (top) and histogram for the migrated cell number from three independent experiments (bottom) in the 3 h transwell assay for ACHN cells in the presence of the designated recombinant human LTF (rec hLTF) protein. In (**E**,**G**), Kruskal–Wallis test was use to estimate the statistical significance of three independent experiments. The symbol “***” denotes the statistical significance at *p* < 0.001. (**H**) Western blot analysis for the protein levels of p-Akt, Akt, p-mTOR, mTOR and GAPDH in ACHN cells treated with various rec hLTF concentrations for 2 h. In (**F**,**H**), GAPDH was used as an internal control of protein loading.

In our previous report [24], we have demonstrated that a low-level LTF expression is associated with a high risk for cancer metastasis and poor prognosis in TCGA ccRCC patients. To ascertain a possible mechanism underlying the metastatic progression of ccRCC with LTF downregulation, here we firstly performed a computational simulation using the Gene Set Enrichment Analysis (GSEA) program. Prior to the simulation, we generated two LTF-related gene sets derived from the TCGA ccRCC database and GSE36972 dataset. We generated one of LTF-related gene sets by performing LTF coexpression status that was ranked by Spearman’s correlation test with other somatic genes included in RNA sequencing experiment against primary tumors derived from TCGA ccRCC patients stratified as a low LTF-expression group in a Kaplan-Meier analysis [24] and recorded as lymph node metastasis (Figure 1A). On the other hand, we generated the other LTF gene set which was ranked by a log2 fold change of mRNA levels for all somatic genes in the LTF-overexpressing cells as compared to vector control cells in the GSE36972 dataset (Figure 1A). The GSEA simulation revealed that the gene set generated from TCGA ccRCC with a lower LTF expression and lymph node metastasis positively while the gene set from the mRNA fold-change of somatic genes after LTF overexpression in the GSE36972 dataset negatively correlates with the MTORC1 gene set (Figure 1B). Moreover, in the poorly metastatic A498 cells, LTF knockdown predominantly repressed the endogenous LTF mRNA levels (Figure 1C) and suppressed the secreted LTF protein levels (Figure 1D), but dramatically enhanced the cellular migration ability (Figure 1E) and the phosphorylation Akt/mTOR proteins (Figure 1F). Conversely, the inclusion of recombinant human LTF protein concentration-dependently inhibited the cellular migration ability (Figure 1G) and the phosphorylation Akt/mTOR proteins (Figure 1H) in the highly metastatic ACNH cells. Similar views were also found in the other metastatic ccRCC cell line Caki-1 (Supplementary Materials Figure S1A,B).

**Figure 2:**
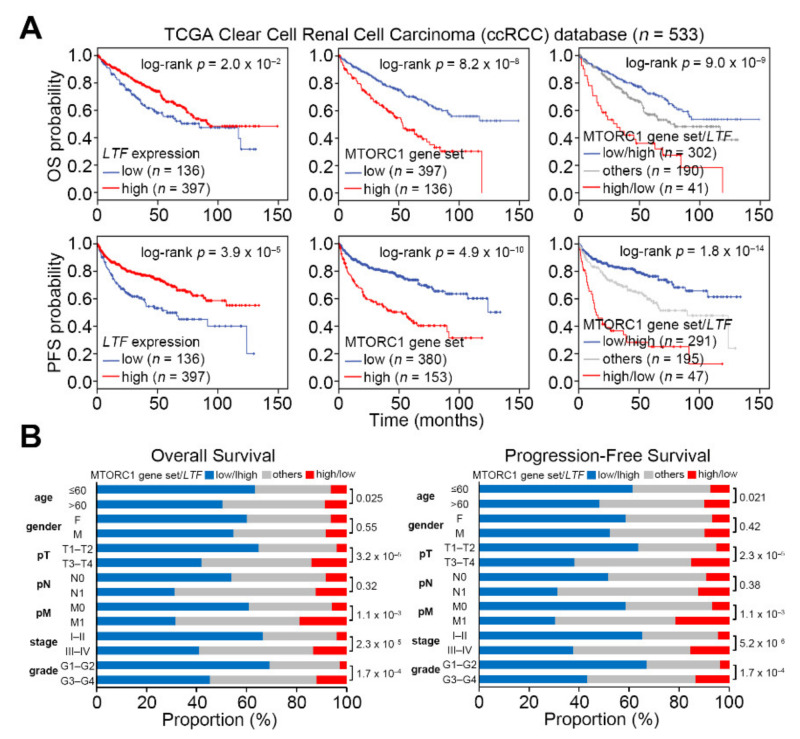
The signature of combining an upregulated mTORC1 activity and downregulated LTF level predicts a worse prognosis and associates with the metastatic progression in ccRCC. (**A**) Kaplan–Meier analyses against the individual or combined mRNA levels of LTF and MTORC1 gene set under the overall survival (OS, upper panel) and progression-free survival (PFS, lower panel) probability. The stratification was performed under a minimized log-rank *p* value. (**B**) Fisher’s exact test for the combined signature of MTORC1 gene set and LTF mRNA levels and pathologic variables including age, gender, pT, pN, pM, stage and grade from the TCGA ccRCC database. The signature “others” represents the low/low and high/high mRNA levels of MTORC1 gene set and LTF.

Kaplan–Meier analyses demonstrated that a lower level of LTF transcript but a higher mRNA level of the mTORC1 gene set was associated with the poorer overall and progression-free survival probabilities in the TCGA ccRCC cohort (Figure 2A). Notably, another Kaplan–Meier analysis revealed that the signature of combining a low-level LTF with a high-level mTORC1 gene set expression predicted worse prognosis under overall or progression-free survival condition in TCGA ccRCC patients (Figure 2A). Furthermore, the chi-square test showed that the proportion for the signature of combining a low-level LTF with a high-level mTORC1 gene set expression according to the stratification under either overall or progression-free survival condition is relatively higher in ccRCC patients over 60 years of age, advanced pathologic T status (T3 and T4), distant metastasis (M1), advance pathologic stage (III and IV) or higher neoplasm grade (G3 and G4) (Figure 2B). These findings suggest that LTF downregulation might restore mTORC1 activity to promote the metastatic progression of ccRCC.

### 3.2. LTF Promotes Cellular Migration Ability via Triggering Autophagy Formation in ccRCC

**Figure 3:**
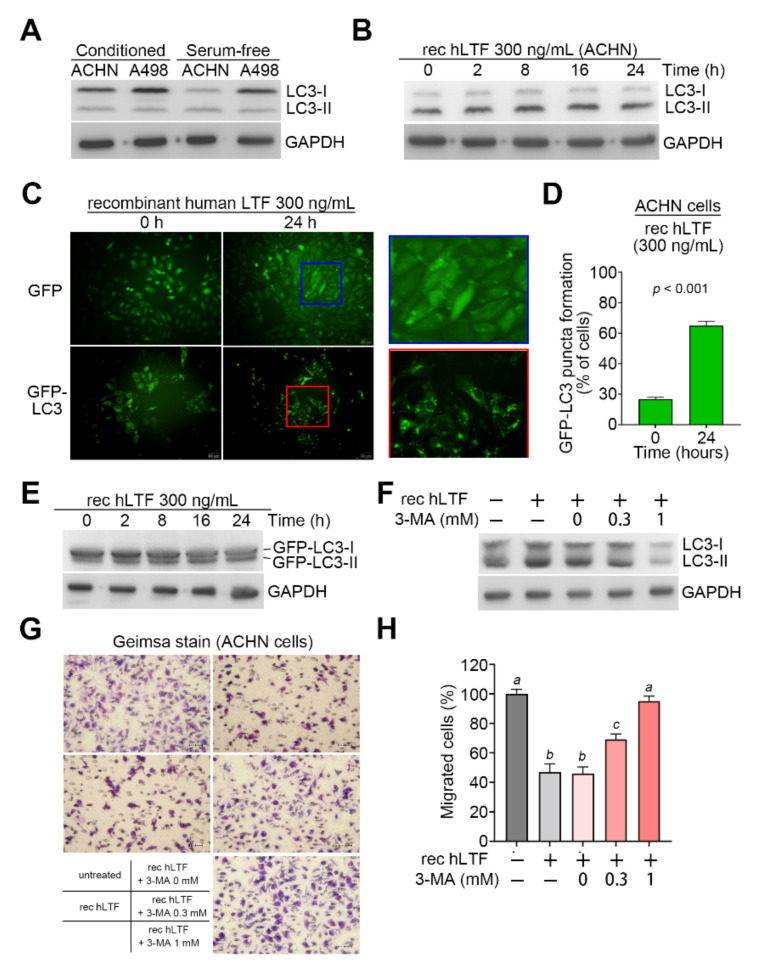
LTF expression modulates autophagy initiation in ccRCC cells. (**A**,**B**) Western blot analyses for LC3I/II and GAPDH proteins in whole cell lysates derived from the ACHN and A498 cells (**A**) and ACHN cells treated with rec hLTF at 300 ng/mL for the indicated time intervals (**B**). (**C**) The fluorescent microscopic observation of ACHN cells that were stably transfected with gene encoding GFP or GFP-LC3 fusion protein cultivated in the presence of rec hLTF at 300 ng/mL on 0 and 24 h. Two representative pictures captured from GFP (blue region) and GFP-LC3 (red region)-expressing ACHN cells were used to highlight the punctate distribution of autophagosomes in GFP-LC3-expressing, not GFP-expressing, ACHN cells. (**D**) The histogram represents the percentage for the punctate formation of autophagosomes in GFP-LC3-expressing ACHN cells treated with rec hLTF at 300 ng/mL for 0 and 24 h. Error bars represent the mean ± SEM of data obtained from three independent experiments and the statistical differences were analyzed by Mann–Whitney test. (**E**) Western blot analyses for GFP-LC3-I/II and GAPDH proteins in whole cell lysates derived from GFP-LC3-expressing ACNH cells treated rec hLTF at 300 ng/mL for the indicated time intervals. (**F**) Western blot analyses for LC3-I/II and GAPDH proteins levels in the untreated ACHN cells and ACHN cells treated with rec hLTF at 300 ng/mL for 24 h combined without or with the pretreatment with 3-MA at the designated concentrations for 1 h. In (**A**,**B**,**E**,**F**), GAPDH was used as an internal control of protein loading. (**G**,**H**) Giemsa staining (**G**) and cell number (**H**) of the migrated ACNH cells in the designated treatments. Data obtained from three independent experiments are presented as the mean ± SEM. Alphabets a, b and c indicate the significant differences at *p* < 0.01 analyzed by nonparametric Kruskal–Wallis test.

Our previous research has shown that LTF induced autophagy in human kidney proximal tubular cells [26]. Furthermore, previous reports demonstrated that Akt/mTOR pathway is able to negatively regulate the formation of autophagy [27,28]. Hence, we measured the levels of LC3-II which is a phosphatidylethanolamine-conjugated form of LC3-I and acts as a key initiator for autophagy formation. Western blot analyses showed that the protein levels of LC3-I are relatively lower in the highly metastatic ACHN cells compared to the poorly metastatic A498 cells cultivated in either conditioned or serum-free media (Figure 3A). Moreover, the inclusion of recombinant human LTF dramatically elevated the levels of LC3-II in ACHN cells (Figure 3B) and Caki-1 cells (Supplementary Materials Figure S1C). To visualize the autophagosome formation, we further stably transfected the construct harboring the GFP or GFP-LC3 fusion gene into ACHN cells. The fluorescent microscopic observation showed that the incubation with recombinant human LTF for 24 h significantly (*p* < 0.001) enhanced the formation of autophagosome as judged by the increased GFP-LC3 puncta formation in the ACHN cells stably transfected with the GFP-LC3 fusion gene (Figure 3C,D). Accordingly, a Western blot analysis showed that the addition of recombinant human LTF time-dependently elevated the protein levels of GFP-LC3-II in ACHN cells (Figure 3E). Robustly, the pretreatment with autophagy inhibitor 3-methyadenine (3-MA) suppressed the intracellular protein levels of LC3-II but rescued the cellular migration ability of ACHN cells in a dose-dependent manner (Figure 3F–H). 

**Figure 4:**
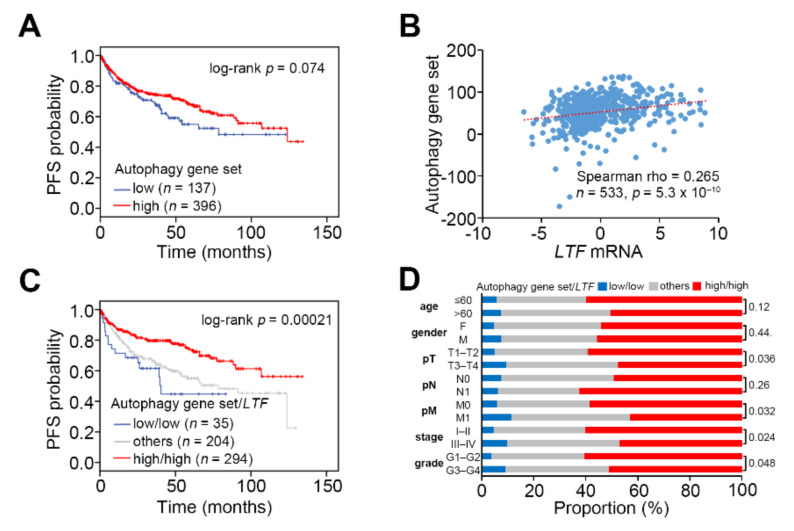
LTF downregulation combined with a reduced autophagy activity correlates with a worse prognosis in ccRCC patients. (**A**) Kaplan–Meier analyses for autophagy gene set mRNA levels using progression-free survival probability in TCGA ccRCC patients. The stratification was performed under a minimized log-rank *p* value. (**B**) Scatchard plot for LTF and autophagy gene set mRNA levels from the TCGA ccRCC database. Spearman’s correlation test was used to determine the statistical significance of LTF and autophagy gene set coexpression in TCGA ccRCC. (**C**) Kaplan-Meier analyses for the combined autophagy gene set and LTF mRNA levels under progression-free survival condition in TCGA ccRCC patients. (**D**) Fisher’s exact test for the signature of combining autophagy gene set and LTF mRNA levels and pathologic variables including age, gender, pT, pN, pM, stage and grade from the TCGA ccRCC database. The signature “others” denotes the low/high and high/low mRNA levels of autophagy gene set and LTF.

Under the progression-free survival condition, Kaplan–Meier analysis revealed that a low-level autophagy gene set expression associated with poor prognosis in the TCGA ccRCC cohort even though there was no statistical significance (Figure 4A). A Spearman correlation test indicated that the mRNA levels of LTF and autophagy gene set were positively correlated in the primary tumors derived from TCGA ccRCC patients (Figure 4B). Significantly, another Kaplan-Meier analysis indicated that the signature of combining low-level LTF and autophagy gene set refers to a poorer progression-free survival probability in TCGA ccRCC patients (Figure 4C). Besides, the Fisher’s exact test revealed that this signature was extensively detected in ccRCC derived from patients who were recorded as having a higher pathologic T status (T3 and T4), pathologic M1, higher pathologic stage (III and IV) or higher neoplasm grade (G3 and G4) (Figure 4D). 

### 3.3. LTF Downregulation Combined with an Enhanced mTORC1 Activity Serves as an Independent Factor to Predict Cancer Progression in ccRCC Patients

**Figure 5:**
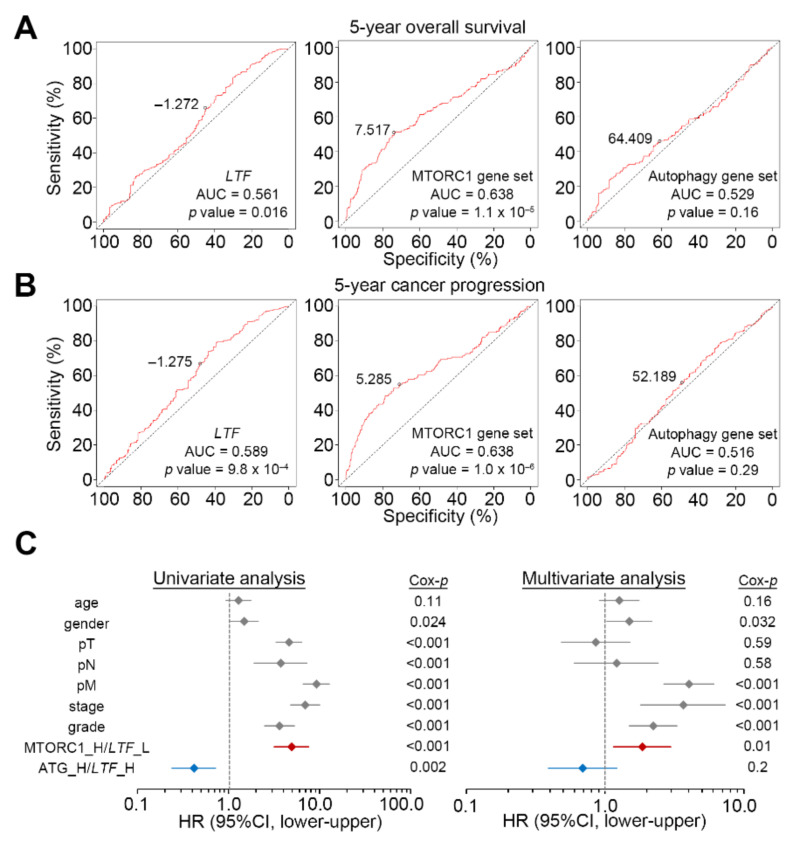
The signature of combining an upregulated mTORC1 activity and downregulated LTF level is an independent prognostic biomarker in ccRCC. (**A**,**B**) the receiver operating characteristic (ROC) plot for the mRNA levels of LTF, MTORC1 gene set and autophagy gene set against the 5-year overall survival rate (**A**) and cancer progression probability (**B**) using TCGA ccRCC database. The AUC is the abbreviation of area under the curve. The dashed lines indicate the nonpredictive value. The inserted values denote the strongest cutoff of mRNA levels for LTF, MTORC1 gene set and autophagy gene set in the ROC analyses to discriminate 5-year survival and cancer progression. (**C**) Cox regression test using univariate (left) and multivariate (right) modes for pathological variables including age (median = 60 years, elder vs. younger), gender (male vs. female), pathologic T (pT, T1/T2 vs. T3/T4), pN (N1 vs. N0), pM (M1 vs. M0), stage (III/IV vs. I/II) and grade (III/IV vs. I/II) and the mRNA levels of MTORC1/LTF (high/low vs. low/high) autophagy gene set/LTF (low/low vs. high/high) under the condition of progression-free survival probability for TCGA ccRCC patients.

To understand the predictive value of LTF, MTORC1 gene set and autophagy for 5-year overall survival and cancer progression, we next performed receiver operating characteristic (ROC) analyses against the TCGA ccRCC cohort. The data showed that compared to the LTF and autophagy gene set, the MTORC1 gene set acted as a stronger predictive biomarker for 5-year overall survival (Figure 5A) and cancer progression (Figure 5B). Importantly, in comparison with the combination of low-level LTF and autophagy gene set, as well as other clinicopathological confounders, the signature of combining the low-level LTF and high-level MTORC1 gene set appears to be an independent prognostic factor in the multivariate analysis of a Cox regression test (Figure 5C).

**Figure 6:**
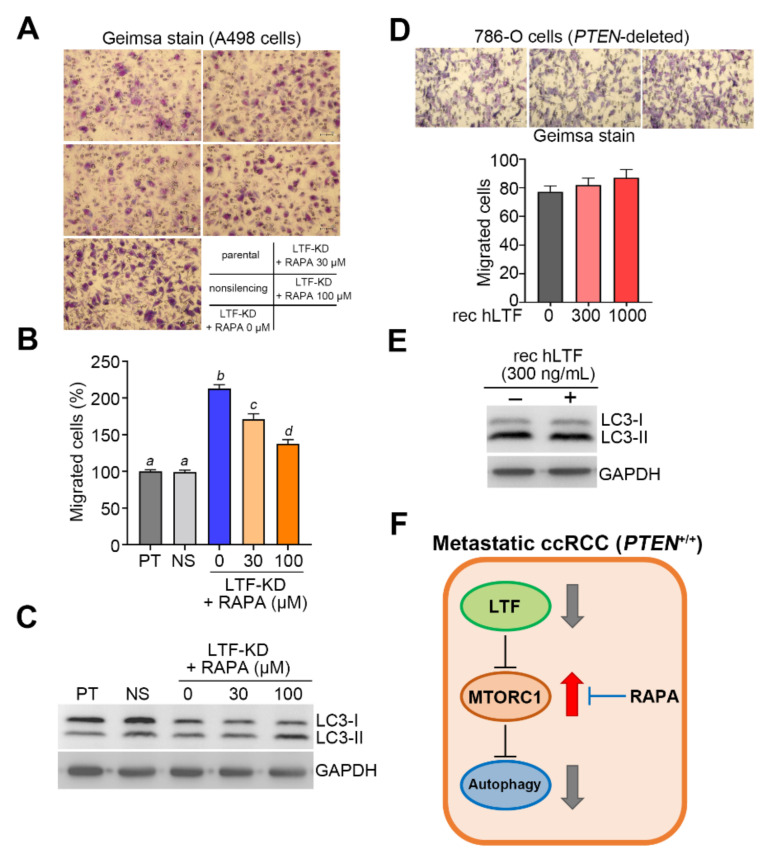
Pharmaceutical inhibition of mTORC1 activity by rapamycin restores autophagy function but compromises the cellular ability of LTF-silencing A498 cells. (**A**,**B**) Giemsa staining (**A**) and the migrated cell number (**B**) in 3 h transwell cultivation for the parental and nonsilencing control A498 cells and LTF-KD A498 cells pretreated with rapamycin (RAPA) at the designated concentrations for 24 h. Data obtained from three independent experiments are presented as the mean ± SEM. Alphabets a, b and c indicate significant differences at *p* < 0.01 analyzed by nonparametric Kruskal–Wallis test. (**C**) Western blot analysis for LC3I/II and GAPDH proteins in whole cell lysates derived from parental and nonsilencing control A498 cells and LTF-KD A498 cells pretreated with rapamycin (RAPA) at designated concentrations for 24 h. (**D**) Giemsa staining for the migrated cells (top) and histogram for the migrated cell number from three independent experiments (bottom) in the 3 h transwell assay for 786-O cells in the presence of the designated recombinant human LTF (rec hLTF) protein. (**E**) Western blot analyses for LC3I/II and GAPDH proteins in whole cell lysates derived from the 786-O cells treated without or with rec hLTF at 300 ng/mL for 8 h. (**F**) The illustration for the status of LTF expression and the activities of mTORC1 and autophagy in the metastatic progression of ccRCC.

To evaluate the therapeutic effectiveness of targeting mTOR activity on the metastatic ccRCC with LTF downregulation, we next cultivated A498 cells with LTF knockdown in the absence or presence of mTOR inhibitor rapamycin (RAPA). The data showed that the addition of RAPA dose-dependently suppressed the enhanced cellular migration ability due to LTF knockdown (Figure 6A,B) but reinforced the protein levels of LC3-II (Figure 6C) in A498 cells. Because *PTEN* is an upstream regulator of Akt/mTOR pathway and frequently mutated in ccRCC, we next employed the *PTEN*-deleted ccRCC cell line 786-O to evaluate the LTF effects. The data showed that the addition of recombinant human LTF did not affect the cellular migration ability (Figure 6D) and the protein levels of LC3-I/II (Figure 6E) in 786-O cells. These findings suggest that therapeutic targeting of mTOR activity by its inhibitors, e.g., RAPA, could be a good strategy to combat the metastatic ccRCC with wild-type *PTEN* and LTF downregulation (Figure 6F).

## 4. Discussion

It has been shown that approximate 20% of ccRCC patients are frequently diagnosed at the metastatic stage and the 5-year overall survival rate is about 40% [29]. Recently, nonspecific immunotherapy with high-dose interleukin-2 and novel immune checkpoint inhibitors have been employed to treat metastatic ccRCC. In addition, the inhibitors (sunitinib and pazopanib) against the tyrosine kinase activity of vascular endothelial growth factor (VEGF) are the most effective first-line options for patients with metastatic ccRCC [30]. In the poor-risk patients, the combination of dual checkpoint inhibitors nivolumab and ipilimumab was considered as the preferred first-line therapy [31,32]. In addition to VEGF and immune checkpoints inhibitors, mTOR inhibitors were also used to combat metastatic RCC [33]. Indeed, the mTOR signaling axis was found to be activated in approximately 60% of ccRCC [34]. In fact, the United States Food and Drug Administration and the European Medicines Agency have approved the mTOR inhibitor temsirolimus as a first-line to treat metastatic ccRCC in 2007 and temsirolimus is listed as a category 1 drug for front-line treatment of poor-risk patients in the National Comprehensive Cancer Network (NCCN) Kidney Cancer Panel, which is based on the phase III NCT0065468 trial for temsirolimus. In this trial, patients receiving temsirolimus achieved longer progression-free and overall survival [35]. Nevertheless, identifying better biomarkers and predictive models used in patient selection to increase response is urgently needed. In this study, our data show that LTF downregulation is accompanied with an enhanced activity of the mTORC1 pathway in metastatic ccRCC. This finding suggests that metastatic ccRCC patients with LTF deficiency might be sensitive to temsirolimus therapy. 

Previous reports have demonstrated that LTF downregulation refers to a poor 5-year survival rate in breast cancer patients [36] and correlates with the biochemical recurrence in the hormone-resistant prostate cancer patients receiving radical prostatectomy [37]. Besides, we recently reported that LTF downregulation drove the metastatic progression of ccRCC [24]. Here, we show that the signature of combining low-level LTF and a high-level MTORC1 gene set at their mRNA expression correlates with a worse progression-free survival condition and serves as an independent prognostic factor for cancer progression in a multivariate analysis against clinicopathological confounders and the combination of LTF and autophagy gene set mRNA levels in ccRCC patients. According to a Kaplan–Meier analysis, approximately 9% of ccRCC patients were stratified as low-level LTF and high-level MTORC1 gene set expression using progression-free survival probability and had a shortest time period for cancer progression after the first treatment. Nevertheless, this subpopulation of ccRCC patients might be sensitive to targeted therapy against mTOR activity. Indeed, our results showed that LTF knockdown robustly promoted the metastatic potentials of ccRCC cells but conversely rendered those cells sensitive to the treatment of mTOR inhibitor rapamycin. Similar views were also found in a previous report that LTF inhibited cell growth of highly metastatic triple-negative breast cancer cell lines MDA-MB231 and Hs578t via suppressing the mTOR activity [38].

The requirement of autophagy activity during cancer metastasis is still controversial. It has been reported that autophagy activity is required for cancer metastasis [39]; however, several studies have also claimed that autophagy formation is restrained during the metastatic progression in the different types of cancer [40, 41 and 42]. In RCC cells, autophagy induction was found to promote epithelial–mesenchymal transition and cellular invasion ability [43,44]; however, the opposite views were also reported in other studies [45,46]. In this study, we showed that the restrained autophagy initiation was detected in highly metastatic ccRCC cells with LTF downregulation. Therefore, further studies are needed to elucidate whether the discrepancy is associated with LTF expression in metastatic RCC.

## 5. Conclusions

Our results demonstrated that LTF downregulation triggers the metastatic progression in ccRCC via recruiting the activity of the Akt/mTORC1 pathway. Because the mTOR inhibitor temsirolimus has been approved as a first-line drug to treat metastatic ccRCC, LTF downregulation may serve as a good biomarker to guide the medication of temsirolimus in clinics to treat metastatic ccRCC without *PTEN* mutation. However, further experiment regarding the restoration of functional *PTEN* expression in 786-O cells is needed to validate the negative regulation of the Akt/mTOR pathway by the LTF–*PTEN* signaling axis.

## Data Availability

The data presented in this study are available in the article.

## Supplementary Materials

The following are available online at www.mdpi.com/article/10.3390/biomedicines9121896/s1, Figure S1: LTF addition suppresses the cellular migration ability of Caki-1 cells via inhibiting the activity pf Akt/mTOR pathway and enhancing the autophagy formation. Figure S2: Uncut blots for Figure 1F,H, Figure 3A,B,E,F and Figure 6C. Figure S3: Uncut blots for Figure 6E, Figure S1B,C.

## References

[1] Rini B.I., Campbell S.C., Escudier B. (2009). Renal cell carcinoma. Lancet.

[2] Frank I., Blute M.L., Cheville J.C., Lohse C.M., Weaver A.L., Zincke H. (2002). An outcome prediction model for patients with clear cell renal cell carcinoma treated with radical nephrectomy based on tumor stage, size, grade and necrosis: The SSIGN score. J. Urol..

[3] Motzer R.J., Hutson T.E., McCann L., Deen K., Choueiri T.K. (2014). Overall survival in renal-cell carcinoma with pazopanib versus sunitinib. N. Engl. J. Med..

[4] Fillebeen C., Ruchoux M.M., Mitchell V., Vincent S., Benaissa M., Pierce A. (2001). Lactoferrin is synthesized by activated microglia in the human substantia nigra and its synthesis by the human microglial CHME cell line is upregulated by tumor necrosis factor alpha or 1-methyl-4-phenylpyridinium treatment. Brain Res. Mol. Brain Res..

[5] Luo G., Zhou Y., Yi W., Yi H. (2015). Lactotransferrin expression is downregulated and affects the mitogen-activated protein kinase pathway in gastric cancer. Oncol. Lett..

[6] Zhou Y., Zeng Z., Zhang W., Xiong W., Wu M., Tan Y., Yi W., Xiao L., Li X., Huang C. (2008). Lactotransferrin: A candidate tumor suppressor-Deficient expression in human nasopharyngeal carcinoma and inhibition of NPC cell proliferation by modulating the mitogen-activated protein kinase pathway. Int. J. Cancer.

[7] Farnaud S., Evans R.W. (2003). Lactoferrin—A multifunctional protein with antimicrobial properties. Mol. Immunol..

[8] Fernandes K.E., Carter D.A. (2017). The Antifungal Activity of Lactoferrin and Its Derived Peptides: Mechanisms of Action and Synergy with Drugs against Fungal Pathogens. Front. Microbiol..

[9] Lu J., Haley K.P., Francis J.D., Guevara M.A., Doster R.S., Craft K.M., Moore R.E., Chambers S.A., Delgado A.G., Piazuelo M.B. (2021). The Innate Immune Glycoprotein Lactoferrin Represses the Helicobacter pylori cag Type IV Secretion System. CheBioChem.

[10] Al-Mogbel M.S., Menezes G.A., Elabbasy M.T., Alkhulaifi M.M., Hossain A., Khan M.A. (2021). Effect of Synergistic Action of Bovine Lactoferrin with Antibiotics on Drug Resistant Bacterial Pathogens. Medicina.

[11] Allaire A., Picard-Jean F., Bisaillon M. (2015). Immunofluorescence to Monitor the Cellular Uptake of Human Lactoferrin and its Associated Antiviral Activity Against the Hepatitis C Virus. J. Vis. Exp..

[12] Campione E., Cosio T., Rosa L., Lanna C., Di Girolamo S., Gaziano R., Valenti P., Bianchi L. (2020). Lactoferrin as Protective Natural Barrier of Respiratory and Intestinal Mucosa against Coronavirus Infection and Inflammation. Int. J. Mol. Sci..

[13] Hering N.A., Luettig J., Krug S.M., Wiegand S., Gross G., van Tol E.A., Schulzke J.D., Rosenthal R. (2017). Lactoferrin protects against intestinal inflammation and bacteria-induced barrier dysfunction in vitro. Ann. N. Y. Acad. Sci..

[14] Chen J.M., Fan Y.C., Lin J.W., Chen Y.Y., Hsu W.L., Chiou S.S. (2017). Bovine Lactoferrin Inhibits Dengue Virus Infectivity by Interacting with Heparan Sulfate, Low-Density Lipoprotein Receptor, and DC-SIGN. Int. J. Mol. Sci..

[15] Shi H., Li W. (2014). Inhibitory effects of human lactoferrin on U14 cervical carcinoma through upregulation of the immune response. Oncol. Lett..

[16] Zhang Y., Lima C.F., Rodrigues L.R. (2014). Anticancer effects of lactoferrin: Underlying mechanisms and future trends in cancer therapy. Nutr. Rev..

[17] Cutone A., Ianiro G., Lepanto M.S., Rosa L., Valenti P., Bonaccorsi di Patti M.C., Musci G. (2020). Lactoferrin in the Prevention and Treatment of Intestinal Inflammatory Pathologies Associated with Colorectal Cancer Development. Cancers.

[18] Kondapi A.K. (2020). Targeting cancer with lactoferrin nanoparticles: Recent advances. Nanomedicine.

[19] Ryu M., Nogami A., Kitakaze T., Harada N., Suzuki Y.A., Yamaji R. (2017). Lactoferrin induces tropoelastin expression by activating the lipoprotein receptor-related protein 1-mediated phosphatidylinositol 3-kinase/Akt pathway in human dermal fibroblasts. Cell Biol. Int..

[20] Damiens E., El Yazidi I., Mazurier J., Duthille I., Spik G., Boilly-Marer Y. (1999). Lactoferrin inhibits G1 cyclin-dependent kinases during growth arrest of human breast carcinoma cells. J. Cell Biochem..

[21] Pereira C.S., Guedes J.P., Goncalves M., Loureiro L., Castro L., Geros H., Rodrigues L.R., Corte-Real M. (2016). Lactoferrin selectively triggers apoptosis in highly metastatic breast cancer cells through inhibition of plasmalemmal V-H+-ATPase. Oncotarget.

[22] Chea C., Miyauchi M., Inubushi T., Okamoto K., Haing S., Nguyen P.T., Imanaka H., Takata T. (2018). Bovine lactoferrin reverses programming of epithelial-to-mesenchymal transition to mesenchymal-to-epithelial transition in oral squamous cell carcinoma. Biochem. Biophys. Res. Commun..

[23] Jiang R., Lonnerdal B. (2017). Bovine lactoferrin and lactoferricin exert antitumor activities on human colorectal cancer cells (HT-29) by activating various signaling pathways. Biochem. Cell Biol..

[24] Chiu I.J., Hsu Y.H., Chang J.S., Yang J.C., Chiu H.W., Lin Y.F. (2020). Lactotransferrin Downregulation Drives the Metastatic Progression in Clear Cell Renal Cell Carcinoma. Cancers.

[25] Runwal G., Stamatakou E., Siddiqi F.H., Puri C., Zhu Y., Rubinsztein D.C. (2019). LC3-positive structures are prominent in autophagy-deficient cells. Sci Rep..

[26] Hsu Y.H., Chiu I.J., Lin Y.F., Chen Y.J., Lee Y.H., Chiu H.W. (2020). Lactoferrin Contributes a Renoprotective Effect in Acute Kidney Injury and Early Renal Fibrosis. Pharmaceutics.

[27] Li D., Dai L., Wang L., Tan C., Cai J., Shen H., Zhang T., Zhi S., Yang Z., Hu Y. (2021). Novel sophoridine derivatives induce apoptosis and autophagy to suppress the growth of triple-negative breast cancer through inhibition of mTOR signaling. ChemMedChem.

[28] Ji Y., Hu W., Jin Y., Yu H., Fang J. (2021). Liquiritigenin exerts the anti-cancer role in oral cancer via inducing autophagy-related apoptosis through PI3K/AKT/mTOR pathway inhibition in vitro and in vivo. Bioengineered.

[29] Ljungberg B., Campbell S.C., Choi H.Y., Jacqmin D., Lee J.E., Weikert S., Kiemeney L.A. (2011). The epidemiology of renal cell carcinoma. Eur. Urol..

[30] Motzer R.J., Bukowski R.M. (2006). Targeted therapy for metastatic renal cell carcinoma. J. Clin. Oncol..

[31] Powles T., Albiges L., Staehler M., Bensalah K., Dabestani S., Giles R.H., Hofmann F., Hora M., Kuczyk M.A., Lam T.B. (2017). Updated European Association of Urology Guidelines Recommendations for the Treatment of First-line Metastatic Clear Cell Renal Cancer. Eur. Urol..

[32] Heng D.Y., Xie W., Regan M.M., Harshman L.C., Bjarnason G.A., Vaishampayan U.N., Mackenzie M., Wood L., Donskov F., Tan M.H. (2013). External validation and comparison with other models of the International Metastatic Renal-Cell Carcinoma Database Consortium prognostic model: A population-based study. Lancet Oncol..

[33] Hudes G.R. (2007). mTOR as a target for therapy of renal cancer. Clin. Adv. Hematol. Oncol..

[34] Robb V.A., Karbowniczek M., Klein-Szanto A.J., Henske E.P. (2007). Activation of the mTOR signaling pathway in renal clear cell carcinoma. J. Urol..

[35] Hudes G., Carducci M., Tomczak P., Dutcher J., Figlin R., Kapoor A., Staroslawska E., Sosman J., McDermott D., Bodrogi I. (2007). Temsirolimus, interferon alfa, or both for advanced renal-cell carcinoma. N. Engl. J. Med..

[36] Naleskina L.A., Lukianova N.Y., Sobchenko S.O., Storchai D.M., Chekhun V.F. (2016). Lactoferrin expression in breast cancer in relation to biologic properties of tumors and clinical features of disease. Exp. Oncol..

[37] Shaheduzzaman S., Vishwanath A., Furusato B., Cullen J., Chen Y., Banez L., Nau M., Ravindranath L., Kim K.H., Mohammed A. (2007). Silencing of Lactotransferrin expression by methylation in prostate cancer progression. Cancer Biol. Ther..

[38] Zhang Y., Nicolau A., Lima C.F., Rodrigues L.R. (2014). Bovine lactoferrin induces cell cycle arrest and inhibits mTOR signaling in breast cancer cells. Nutr. Cancer.

[39] Mowers E.E., Sharifi M.N., Macleod K.F. (2017). Autophagy in cancer metastasis. Oncogene.

[40] Zhang M., Liu S., Chua M.S., Li H., Luo D., Wang S., Zhang S., Han B., Sun C. (2019). SOCS5 inhibition induces autophagy to impair metastasis in hepatocellular carcinoma cells via the PI3K/Akt/mTOR pathway. Cell Death. Dis..

[41] Zhao G.S., Gao Z.R., Zhang Q., Tang X.F., Lv Y.F., Zhang Z.S., Zhang Y., Tan Q.L., Peng D.B., Jiang D.M. (2018). TSSC3 promotes autophagy via inactivating the Src-mediated PI3K/Akt/mTOR pathway to suppress tumorigenesis and metastasis in osteosarcoma, and predicts a favorable prognosis. J. Exp. Clin. Cancer Res..

[42] Zhu J.F., Huang W., Yi H.M., Xiao T., Li J.Y., Feng J., Yi H., Lu S.S., Li X.H., Lu R.H. (2018). Annexin A1-suppressed autophagy promotes nasopharyngeal carcinoma cell invasion and metastasis by PI3K/AKT signaling activation. Cell Death. Dis..

[43] Singla M., Bhattacharyya S. (2017). Autophagy as a potential therapeutic target during epithelial to mesenchymal transition in renal cell carcinoma: An in vitro study. Biomed. Pharmacother..

[44] Wu C.Z., Zheng J.J., Bai Y.H., Xia P., Zhang H.C., Guo Y. (2018). HMGB1/RAGE axis mediates the apoptosis, invasion, autophagy, and angiogenesis of the renal cell carcinoma. Onco. Targets Ther..

[45] Li F., Ma Z., Guan Z., Chen Y., Wu K., Guo P., Wang X., He D., Zeng J. (2015). Autophagy induction by silibinin positively contributes to its anti-metastatic capacity via AMPK/mTOR pathway in renal cell carcinoma. Int. J. Mol. Sci..

[46] Liu S., Xie F., Wang H., Liu Z., Liu X., Sun L., Niu Z. (2015). Ubenimex inhibits cell proliferation, migration and invasion in renal cell carcinoma: The effect is autophagy-associated. Oncol. Rep..

