# Supplementary material for: Lactotransferrin Downregulation Serves as a Potential Predictor for the Therapeutic Effectiveness of mTOR Inhibitors in the Metastatic Clear Cell Renal Cell Carcinoma without PTEN Mutation
Source: Biomedicines. 2021 Dec 13;9(12):1896. doi: 10.3390/biomedicines9121896 (PMC8698394; doi:10.3390/biomedicines9121896)
Supplement: Supplementary file 1 [file biomedicines-09-01896-s001.zip › biomedicines-1447967-supplementary.pdf]

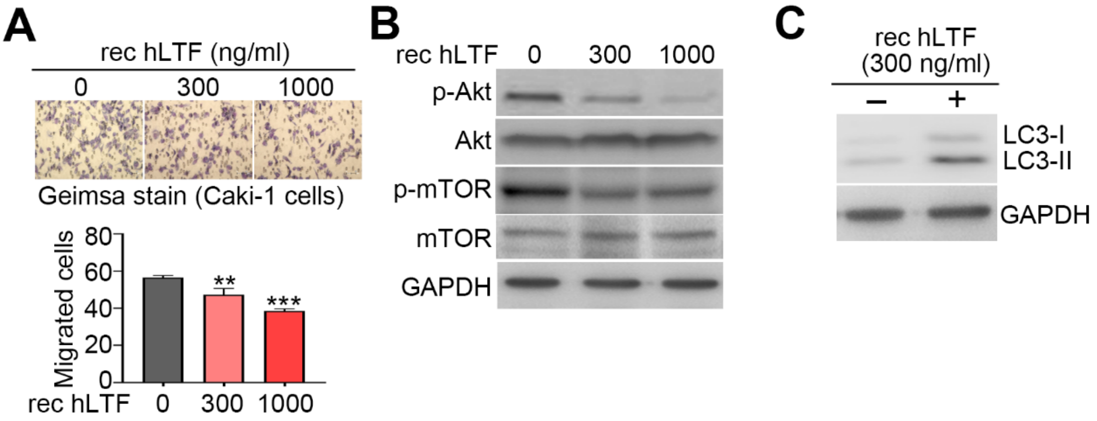


**Figure S1.** LTF addition suppresses the cellular migration ability of Caki-1 cells via inhibiting the activity pf Akt/mTOR pathway and enhancing the autophagy formation. (A) Giemsa staining for the migrated cells (Top) and histogram for the migrated cell number from three independent experiments (bottom) in the 3-hour transwell assay for Caki-1 cells in the presence of the designated recombinant human LTF (rec hLTF) protein. Kruskal Wallis test was use to estimate the statistical significance of three independent experiments The symbol "**" and "***" denote the statistical significance at $p < 0.01$ and $p < 0.001$, respectively. (B) Western blot analysis for the protein levels of p-Akt, Akt, p-mTOR, mTOR and GAPDH in Caki-1 cells treated with the indicated rec hLTF concentrations for 2 hours. (C) Western blot analyses for LC3I/II and GAPDH proteins in whole cell lysates derived from the Caki-1 cells treated without or with rec hLTF at 300 ng/ml for 8 hours. In B and C, GAPDH was used as an internal control of protein loading.

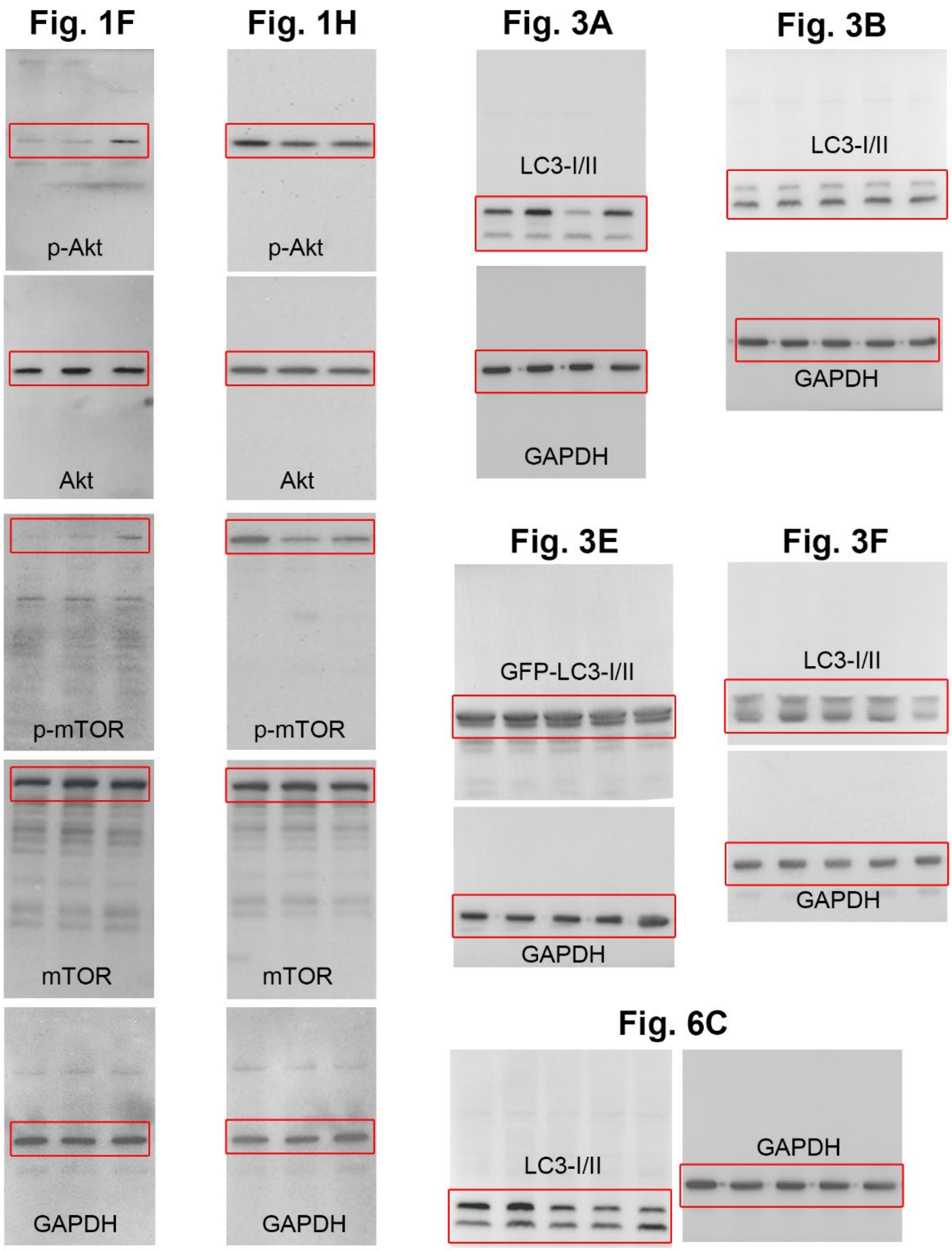


**Figure S2.** Uncut blots for Figure 1F, 1H, 3A, 3B, 3E, 3F and 6C.

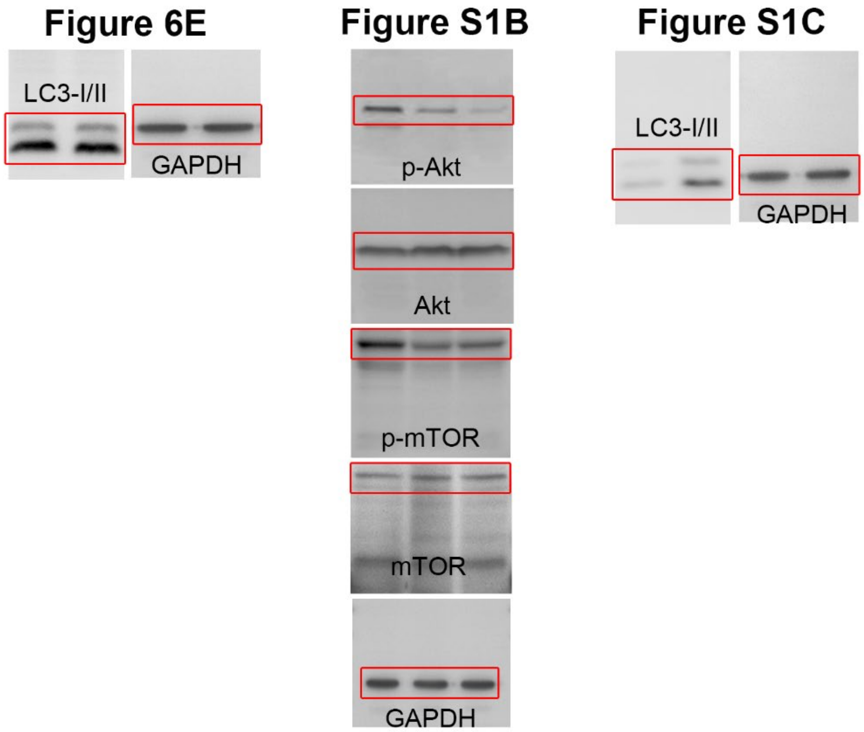


**Figure S3.** Uncut blots for Figure 6E, S1B and S1C.
